# Cultural Distance, Classroom Silence and Culturally Responsive and Inclusive Education: Evidences from Migrant College Students in Shanghai

**DOI:** 10.3390/bs13030193

**Published:** 2023-02-21

**Authors:** Fei Peng, Lili Kang, Jinhai Shi, Ming Liu

**Affiliations:** 1 School of International Economics and Trade, Shanghai Lixin University of Accounting and Finance, No. 995 Shangchuan Road, Shanghai 201209, China; 2 School of Business Administration, Shanghai Lixin University of Accounting and Finance, No. 995 Shangchuan Road, Shanghai 201209, China

**Keywords:** cultural distance, classroom silence, culturally responsive and inclusive education, China, migrant college students

## Abstract

This paper investigated the relationship between cultural distance, classroom silence, and the performance of culturally responsive and inclusive education (CRIE) using a survey of 1051 college students in Shanghai in 2022. We found a significantly positive association between migrant students’ cultural distance and their perceived learning gains in class. Students’ cultural distance increased their classroom silence as a form of protection but had no significant effect on their classroom silence as a sign of power. The classroom silence as protection decreased students’ perceived learning gains. However, classroom silence as power could be used by both local and migrant students as a hold-up strategy to strengthen their influence in class discussions, which could improve their perceived learning gains. Teachers’ CRIE played the most important role in migrant students’ perceived learning gains, while the effectiveness of CRIE was also actually dependent on the different channels and mechanisms of cultural distance and classroom silence. A cautious identification of classroom silence will improve the effectiveness of CRIE. Suggestions are offered to lighten the practice of educators, administrators, and instructors who face classroom silence from subnational migrant students.

## 1. Introduction

Teachers tend to encourage students to talk more in the classroom; however, students’ classroom silence is a pervasive phenomenon around the world. Psychologists, anthropologists, and linguists have endeavored to provide an understanding regarding classroom silence in schools [1,2,3,4,5,6,7]. Bosacki [8] defined silence as the absence of vocalization. Students’ silence is not only a total lack of audible verbalization but also a failure to present a specific subject matter or to say what is requested by teachers. As for classroom silence, students’ silence can be interpreted as a compliant attitude to class order and respect for teachers. On the other hand, it can also be interpreted as resistance to institutional power or self-protection based on knowledge incapability or cultural alienation. Granger [9] regarded classroom silence as increased disobedience, conflict, misbehavior, and even deception, as students were assumed to be listeners. Since classroom silence prevents students’ teachers and peers from assessing their performances, students’ silence can be regarded as a form of protection. 

However, researchers have rarely interpreted students’ silence as an intentional behavioral strategy to gain more attention from teachers and peers. More researchers have regarded silence as part of a continuum, suggesting several different kinds of silence represent a “complex and complicated cultural phenomenon” [6,8]. Silence may bring educational merit in some cases because it enables learning and self-development; however, when it is examined in relation to a student’s academic achievement, it seems to be overwhelmingly evaluated by teachers as an unfavorable phenomenon. Classroom silence may be maintained by students as an emotional resistance against the teacher’s authority and as a way of passively articulating negative emotions. As a psychological state and manner exhibited by a student, classroom silence equates to limited engagement or inadequate involvement in classroom communication. However, at the level of thought, emotion, and action, the absence of talk does not equate to the absence of thought. Thus, educators need to learn to identify and respond to a range of silences that might reflect cultural understandings.

Schultz [5] argued that silence might hold multiple meanings for individuals within and across racial, ethnic, and cultural groups; however, classroom silence may be assigned a limited number of meanings, such as a form of protection or a sign of power. The impact of classroom silence on the productivity of students is dependent on environmental, capability, and cultural difference across student groups. In particular, for those students without access to adequate support and resources, students in particular cultural groups will experience numerous transitions of education and career development throughout their lifespan [10]. Since the works of Philips [11] and Banks [12], more and more attention has been paid to the classroom silence of migrant students from foreign countries and, in particular, cultural groups such as American Indian students, African American students, Japanese American students, and Chinese American girls [5,13,14]. These studies basically used ideologies regarding race diversity in multicultural education; hence, they have been criticized for being “Americano-centric” or “overly Western” by many other authors [15,16,17,18].

Liu et al. [19] pointed out that the studies developed in the Western context were useful for understanding non-Western contexts, as they provided researchers with an initial perspective and a framework to approach the multicultural education field. Following the same vein, another strand of the literature of classroom silence is based on local students learning foreign culture, such as English as a foreign language (EFL) learners in Japan [20], Turkey [21], Australia, China, Korea, and Vietnam [22]. However, Amsler et al. [15] called for multicultural and intercultural education around the world to move “beyond existing frameworks of modernist knowledge, politics and economic systems”. Achieving a profound understanding of the classroom silence of migrant students within a non-Western country is still a complex process, as most migrant students are actually from other regions of the same country, rather than from foreign countries. For instance, more than 200,000 migrant college students are studying in Shanghai [23]. These migrant college students live in host communities far away from their hometowns. Most of them have no social capital and network to gain access to the formal labor market after they graduate and will go back home or move to other cities. It is particularly surprising that the literature rarely includes this silent group. The students, teachers, parents, and researchers, as well as education policymakers call for new methods of conceptualization and evidence-based policies on the classroom silence of migrant students within the same country. Thus, this paper aimed to fill the literature gap by investigating the classroom silence of migrant college students from other regions within a non-Western country, namely, China. 

The academic performance of migrant students moving from rural to urban areas in elementary and secondary schools is well-documented and has been a topic of repeated empirical inquiry in China [24,25,26]. However, studies focusing on students with migration backgrounds at the higher education level are very rare. Therefore, in this paper, we exclusively focused on migrant students in higher education and examined their classroom silence and study performance. The silence of marginal groups of students in classrooms, such as migrant students and cultural minority students, may reflect more transitional unreadiness based on cultural distance and social exclusion. These transitions can result in students exiting education and training programs, unemployment, and mental as well as physical health challenges [10,27]. Migrant students might be reflective, or they may take more time to understand the language with cultural contexture and to put together a response. They may have seriously negative feelings of nervousness, anxiousness, embarrassment, frustration, resistance, and alienation in class and try to use classroom silence as a shelter for the purpose of hiding and self-protection. Students experiencing resistance and alienation find it more difficult to care about learning and to contribute to the collective knowledge of the class. When they frequently choose silence as a way to hide their cultural identities, beliefs, and family background, they lose the opportunity to participate in the classroom and may have inferior performance in terms of their studies. 

Although the classroom silence of migrant students has lately become an area of attention of educators and scholars, further evidence is still required in order to define teachers’ roles in culturally responsive and inclusive education (CRIE). This construct has been regarded as a problem, in terms of the communication between the teachers and students, that not only impacts the completion of the teaching objectives in the classroom but also affects the nurturing of students’ study performance. Teachers’ immediacy behavior, such as stimulating effective educational methods positively, has a noteworthy function in students’ growth and progress [28]. Thus, innovative interventions and studies, especially classroom-based collaboration interventions and research efforts, should be promoted to effectively support CRIE. 

Gay [29,30] developed a CRIE framework grounded in teaching strategies and practices that has five essential components: cultural diversity knowledge, ethnic and cultural diversity curriculum, learning communities, ethnically diverse communication, and culturally diverse instruction. Hence, CRIE is the use of cultural knowledge, past experiences, reference frameworks, and performance styles to increase the efficacy of learning experiences for culturally diverse learners. Samuels [31] and Suárez-Orozco et al. [32] argued that CRIE offers full equitable access to education for students from all cultures and creates an inclusive learning environment in which students feel safe, allowing them to be who they are, and to feel that their contributions and perspectives are valued and respected. As the teaching quality of college students gradually becomes the core issue of higher education in China, the widely observed phenomena of classroom silence has been addressed by researchers, education policy makers, and the general public. At present, the accurate definitions of classroom silence are still unclear in terms of China’s higher education. Teachers in colleges need to identify what types of silence exist in the classroom, what negative consequences those silent students might face, and undoubtedly, what effective pedagogy should be applied to help them, especially when they experience difficulties during their transitional periods.

This paper used a survey of 1051 college students in Shanghai to explore how we can improve the learning gains of migrant college students by better understanding student silence in an educational setting. This paper contributes to the literature in three ways. Firstly, the former literature regarding classroom silence and multicultural education mainly investigated the culture issues of either the migrant students in a foreign Western country or local students as EFL learners [14,33]. We focused on migrant college students from other regions within the same non-Western country—China. We measured the cultural distance of subnational migrant students using the cultivation history of their hometowns in 1916 traditional China (south-rice-north-wheat culture) [34,35,36,37,38,39]. We argued that the behavior of the people who used to reside in some regions with specific cultures exhibits certain psychological patterns. Historical data on cultivation in the agricultural sector can capture exogenous cultural variation across regions [40,41]. The south-rice-north-wheat culture, as the most prominent psychological pattern in China, could affect the participation decision of subnational migrant students in class discussions and, thus, affect their study performance. To the best of our knowledge, this is the first study which considered the effect of subnational migrant students’ cultural distance from the host community on their classroom silence and study performance within a non-Western country. 

Second, in order to explore the relationship between cultural distance, classroom silence, and CRIE, we designed and issued the “The Questionnaire on Classroom Silence in Shanghai 2022”. Based on the common factor analysis (CFA) of 1051 students’ questionnaires, we identified and integrated two types of classroom silence (as a form of protection and as a sign of power) described in the literature, e.g., by Schultz [5]. This empirical division has a high degree of reliability and validity in a statistical sense and can accurately measure different types of classroom silence. Although the accurate identification of the different meanings of classroom silence has been addressed by many authors [8], empirical evidence is surprisingly rare. As far as we know, this is also the first study to place the different meanings of classroom silence into an integrated theoretical framework of cultural distance, CRIE, and study performance of migrant students. Further analysis has shown that these two types of classroom silence have different causes and scenarios, and, therefore, different effects on students’ learning gains. This study highlighted the importance of the identification of the different meanings of classroom silence.

Finally, Au and Mason [42], Erickson and Mohatt [43], Philips [11], Solberg et al. [10], and Park et al. [27] addressed the teacher’s role in creating a culturally responsive and inclusive environment and providing opportunities for migrant students to talk and interact in the classroom. Teachers need to design the participation structures based on cultural differences so that the classroom can become more culturally responsive and inclusive. CRIE is a culturally relevant pedagogy that recognizes the importance of including students’ cultural backgrounds in all aspects of teaching and learning. Migrant students might not have social capital and a network in the host community. They either have no experience in taking advantage of existing opportunities in the classroom. This is particularly important for their teachers to reconsider practices and language in ways that are more inclusive and responsive to their cultural background and prior living experiences [10,27]. We found that the positive effect of CRIE on migrant students’ learning performance was dominant, even though its effectiveness was also actually dependent on the different channels and mechanism of cultural distance and classroom silence. This paper aimed to identify and promote effective pedagogy, based on the empirical evidence, to improve the learning gains of young migrant students. Subsequently, suggestions were offered to lighten the practice of educators, administrators, and instructors.

The remainder of this paper proceeds as follows: based on a review of the related literature, testable hypotheses were developed in Section 2. The empirical model used was specified and explained in Section 3. Section 3 also includes discussion of the data and variables. Empirical results were presented and discussed in Section 4. Section 5 contains a summary of the main results, policy implications, and directions for further research.

## 2. Literature Review and Hypothesis Development

### 2.1. Culture Distance and Migrant Students

Culture in its current Western-centric format is defined as customs, beliefs, and values that can be intergenerationally transferred and are relatively stable in ethnic, religious, or social groups [44,45,46]. Matsumoto et al. [47] point out that, in its broadest sense, culture is the reflection of the way of life of a group of people and is something that is handed down from generation to generation. Stroope [48] and Albert and Trommsdorff [49] consider culture to be the sum of experiences connected to families’ values and traditions, and how one is part of a community. As the result of long-term evolution and manifestation terms of stability and inheritance, culture plays a significant role in shaping the behavior of individuals and can influence the behavior of individuals from childhood, leading to long-lasting patterns in adulthood. 

However, culture in Chinese has strong connotations of change, interaction, and modification [18]. Fang [50] argues that culture is a process of constant change which takes place for one side or both sides concerned, as a result of their contact with each other. Hence, culture as a complex and fluid concept is naturally connected with the broad field of diversity education in China. Birkeland [51] points out that the aims of comparative and international education are to “carry out cultural loans, describe best practices, understand the interrelatedness between education and culture, and develop global solidarity as a world citizen”. Culture in diversity education needs to move beyond cultural essentialism and the all-cultural when comparing educational approaches [52]. Additionally, culture in diversity education needs to move beyond evaluating educational practice from a monocultural perspective which regards one’s group or one’s culture as the best, possessing all the proper answers to problems [18], and instead address the interrelation between globalization and local practices [53]. Thus, in this study, we follow this more up-to-date approach to the concept of culture in education.

Most existing studies have analyzed the impact of cultural distance on the study performance of migrant students at a national level [54,55]. However, in the large country of China, there are more than 30 province-level administrative regions, each of which has a long history and distinctive tradition. Significant cultural differences exist among these regions, so that any Chinese university or college enrolling migrant students from all over the country is actually a “microcosm of Chinese diversity” [18]. Surprisingly, this unique context of intercultural education has been captured by very little research, with the exception of the work of Clothey and Hu [56]. Chinese regional cultures are generally influenced by the geographic characteristics of the natural environment, such as the region’s topography, climate, vegetation, rivers, and soil, as well as the production process and the division of labor modes that originated and evolved in China. The importance of ancient agricultural cultivation in the formation of regional cultures in China was addressed by Talhelm et al. [35], Talhelm et al. [36]; Talhelm [37], Dervin and Yuan [18], Fan et al. [39], Kang et al. [38], and Wang et al. [57]. They argued that different farming modes led to different cultures, symbolized by wheat and rice cultivation in the northern and southern parts of China, respectively. 

In ancient southern China, rice planting was highly dependent on the co-constructing irrigation system and coordinating manpower during the busy farming period, which required social exchanges that resulted in mutual benefits among households within a clan [58,59,60]. Villagers from different families used to invest in each other in the form of gifts and loans to maintain internal networks of collaborative relationships and exclude strangers [61]. A clan based on a lineage of kinship is a closed community that its members identify with and are loyal to. Cooperation within the clan is sustained mainly by moral obligations and reputational incentives that discourage cheating and freeriding [62]. Thus, such collaboration was beneficial for establishing geographical and family networks. 

However, in ancient northern China, wheat planting required temporary employment contracts involving migrant workers to cope with labor shortage problems during the busy wheat seeding and harvesting periods [63]. The interpersonal communication, mutual assistance, and even short-term employment relationships formed general social trust in the ancient agricultural society of the northern wheat growing areas of China. The generalized trust in ancient northern China had a significantly larger radius than that in southern China and allowed migrant workers or strangers to be included in the social network. Such a collaboration mode is not dependent on family ties or biological relationships and features a larger trust radius, including strangers, which is more like Western European society by the 10th century, featuring generalized morality, the absence of kin groups, and cooperation among relatively large populations [64,65]. 

The Yangtze River is used as the line dividing the southern rice cultivation areas and northern wheat cultivation areas in ancient China. Shanghai is the largest and most developed city at the east end of the Yangtze River and the midpoint of the Chinese north–south coast; therefore, it is a perfect anchor city for cultural distance studies. Once the ancient culture evolved into a culture based on different agricultural planting modes, the south-rice-north-wheat culture, which is rooted in the basic psychological differences and differential governance patterns between the northern and southern Chinese populations, could be inherited and carried forward, generation after generation [39,57]. 

For the sake of higher education, culture shapes what we teach, how we teach, and whom we teach in college. Mantiri [66] argued that understanding a student’s culture helps teachers evaluate their own teaching methods and become receptive to diverse learning experiences, therefore playing a crucial role in communicating and receiving information. Educational research focusing on the cultural characteristics of migrant students has two separate lines of reasoning for explaining their learning outcomes. The first argument refers to “immigration optimism”, which addresses migrant-specific resources such as values, culture, optimism, and resilience in influencing educational outcomes [54,67,68,69,70]. The values, culture, optimism, and resilience that migrant students bring with them place them in a better position for educational success than natives. Mishra and Müller [55] also argued that social capital and social networks may explain immigrants’ optimistic educational outcomes. Education has a fundamental role in promoting the integration of students with an immigrant background in host societies. It can help them acquire skills to participate in the economy, promote their social and emotional well-being, and support their participation in the social and civic life of their communities [14]. Thus, migrant students from the farther north or south provinces possess a larger cultural distance and may display a better study performance in terms of their higher education in the most developed city in China. Consequently, we developed our first hypothesis on those migrant students migrating from regions with a different culture:

**H1.**  *Students with a larger cultural distance are more likely to have a better performance in terms of their studies.*

### 2.2. Classroom Silence 

However, the second line of the argument refers to “social background and ethnic disadvantage” [71,72,73] to explain the low academic achievement of students with migration backgrounds. Kristen and Granato [74] also found that language constraints and educational institutional structure interacted with differences in social background, bringing further disadvantages for migrant students who might come from less advanced social backgrounds. The dark side of cultural distance is the social background disadvantages of migrant college students in a strange city [55]. There are challenges in ensuring good outcomes for students with an immigrant background, as they need to overcome more adversities related to displacement, socioeconomic disadvantage, and language barriers [14,75]. Classroom silence can be interpreted as a good sign of compliance, respect, or engagement by teachers. However, it can also be interpreted as a bad sign of a lack of interest, boredom, incapability, resistance, or even hostility, which may have totally different meanings for students. The misunderstanding between teachers and students may bias the classroom silence as the refusal to speak, lack of preparation or knowledge, ignorance, and even a passive move of opposition or hostility, rather than an alternative and optional act of participation. 

Students experiencing resistance and alienation find it more difficult to benefit from class activities. Migrant students might have seriously negative feelings of alienation in class and try to use classroom silence as a form of protection. The argument of “social background and ethnic disadvantage” for migrant students who come from a less advanced social background explains the low academic achievement of students with migration backgrounds based on language constraints, educational institutional structure, and social background differences [74]. When they were constrained by the disadvantages of their cultural identities, beliefs, and family background in a strange city, migrant students were less likely to participate in class activities, and this may result in an inferior performance in terms of their studies. Hence, we produced two hypotheses regarding classroom silence as a form of protection:

**H2a.**  *Students with a larger cultural distance in the classroom are more likely to use classroom silence as a form of protection.*

**H2b.**  *Students using more classroom silence as a form of protection are more likely to display a worse performance in terms of their studies.*

It has been widely observed that the force of words rather than silence frequently wins arguments in class discussions [2,3,4]. Schultz [5], however, also argued that a silent student in a classroom was not necessarily powerless and compliant. Students’ silence can be portrayed as a resistant stance if a person holds onto their silence in protest to the teacher’s power and institutional silencing. The academic, socioemotional, and motivational resilience of students could increase classroom silence, which might be assigned more meaning as a sign of power rather than a form of protection. When the silence preceded the talk, teachers and students’ peers tended to listen more carefully to the statement and think about the discussion issues deeply. Hence, students’ silence has the power to garner attention in classrooms. Bosacki [8] focused on the emotional, spiritual, and sociocultural issues around classroom silence, and addressed the connections between adolescents’ concepts of silence and self-development in their school experiences. Thus, she recommended that the educator with a psychocultural approach to structuring classrooms make use of the positive sides of silence while minimizing the factors that contribute to the negative impact of being silenced. 

Classroom silence, which can be interpreted as a situation where a student refuses to participate in class discussion, or supplies a particularly low level of participation, unless their participation is highly appreciated by the teachers and their classmates, can be a typical hold-up problem in the management literature [76,77,78]. This kind of silence can be a sign of power in situations where the student can influence the teachers and classmates, and even dominate the cultural environment in the classroom. The problem is particularly serious when a class discussion is characterized by complication and uncertainty. A student can use silence to add power to their subsequent words. 

Migrant students with a large cultural distance will experience more difficulties in applying this strategy to deeper class discussions, as they might not fully understand the complicated and uncertain discussion within the cultural context, and, as a result, may not be able to provide a response to guide the direction of discussion. However, if migrant students can properly use classroom silence as a strategy to strengthen their cultural identity and influence class discussions, this can have a significantly positive effect on their study performance. It is not surprising that silence as power can further deepen a difficult class discussion and bring better learning gains in higher education. Harumi [79] found that students’ silence can serve a facilitative function in their success if teachers know how to interact with it. Hanh [80] also demonstrated that the silent behavior of students can promote their learning. Hence, in the same vein of the argument for “social background and ethnic disadvantage”, we constructed the next two hypotheses:

**H3a.**  *Students with a larger cultural distance in the classroom are less likely to use classroom silence as a sign of power.*

**H3b.**  *Students who demonstrate more classroom silence as a sign of power are more likely to display a better performance in terms of their studies.*

### 2.3. Culturally Responsive and Inclusive Education 

The classroom silence of students in marginalized cultural groups highlights teachers’ role in this silencing and draws on the limited meanings of silence. As a result, there is a continuous expectation related to creating participation structures that are inclusive for students and that provide opportunities for students to have more discussion and interaction with teachers and peers [11,42,43]. These participation structures used to be designed with certain cultural groups in mind so that classrooms could become more culturally responsive. The intent of CRIE was to build on students’ cultural strengths and prior experiences rather than the different cultural background that students brought from their hometowns. The traditional goal of CRIE was to increase students talking, which was regarded as a direct proxy of learning. 

However, a new understanding of silence in particular communities, such as those with additional needs or high-risk youth during their transition, has become the focus of more recent research [10,14,27,55,75]. Traditional teaching strategies emphasize a fixed teacher–student relationship in which the teacher is the speaker and provides standardized knowledge and tests, while the student is the listener and receives knowledge. As teachers have more and more diverse classrooms today, the fixed teacher–student relationship has a lack of dynamic and flexible mechanisms to inspire students with different cultural backgrounds or experiences. 

From a justice-oriented view, CRIE is especially pertinent today because educators’ approaches to teaching need to reflect diverse and dynamic cultural backgrounds. Based on Fei’s [81] theory of China as a unified multiethnic country, both education policymakers and scholars in China have developed Chinese CRIE, for example, the “integrated multicultural education” [82,83,84], “national unity education” [85], “Chinese multicultural education” [19], and “unified pluralist education” [18] to promote common economic development, cultural heritage, cross-cultural communication, the political equality of all ethnicity groups, and the ultimate realization of national unity. These policies have some common features in pedagogy. School culture and as many teaching activities as possible should integrate students’ cultural backgrounds and experiences in basic education. Teachers need to promote cultural diversity and respect differences between ethnic/cultural groups by building relationships with their students to ensure they feel respected, valued, and seen for who they are. Building those relationships helps them build community within the classroom and with each other, which is extremely important to allow them to be more tolerant and encouraging to students.

Therefore, it is critical for teachers to identify the cultural meanings of classroom silence and pay attention to the decision-making mechanism of silencing within particular cultural groups at specific times [3,5,8,86,87]. In addition, the meanings of classroom silence constantly shift as they are culturally made and remade over time and in different contexts. CRIE can include the diverse cultures, experiences, and backgrounds of students as channels to a more experimental, inspiring, and integrated learning community. Moreover, CRIE is not only for those migrant students who do not come from local mainstream communities, but it is also an important teaching strategy for the better study performance of both local and migrant students. Hence, we formulated the following hypotheses:

**H4a.**  *Teachers using culturally responsive and inclusive pedagogy can decrease classroom silence.*

**H4b.**  *Teachers using culturally responsive and inclusive pedagogy can improve study performance.*

### 2.4. Methodology 

**Figure 1:**
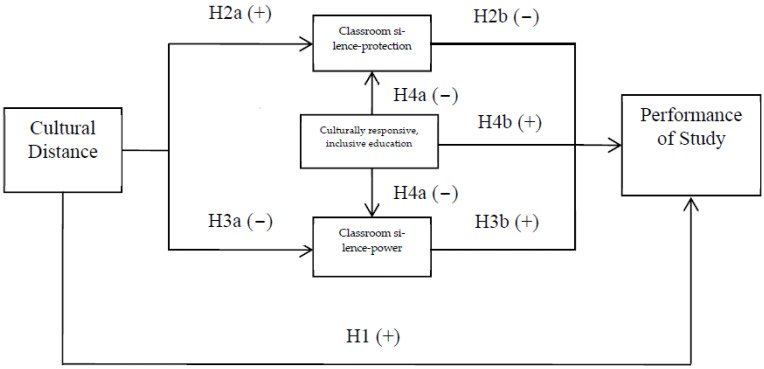
Relationships estimated between cultural distance, classroom silence, CRIE, and performance of students.

In this study, we tested the above hypotheses derived from the literature (see Figure 1) in pursuit of our research objective. The first hypothesis concerned the relationship between cultural distance and study performance and posited that the larger the cultural distance of a migrant student, the better their study performance. The positive relationship between cultural distance and study performance is represented by the *plus* sign: H1(+), while the negative relationship is represented by the *minus* sign (−) in Figure 1. The second set of hypotheses concern the positive association (H2a +) between cultural distance and classroom silence as a form of protection, and the negative association (H2b −) between this kind of silence and study performance. The third set of hypotheses posited that the larger the cultural distance of a migrant student, the lower the possibility that they will use classroom silence as a sign of power (H3a −), which can actually improve their study performance (H3b +). That is, it posited that a cautious identification of classroom silence will be an effective pedagogy.

The fourth set of hypotheses concerned the relationship between CRIE and classroom silence (H4a −), used for protection or power. This hypothesis was advanced to provide a further test of the impact of CRIE on study performance (H4b +). We tested the argument that CRIE would lead to increased classroom interaction, communication, and study performance, but that this outcome would be influenced by the meaning behind the classroom silence. One of advantages of the structural equation model in Figure 1 is the identification of the direct effects and indirect effects of cultural distance and CRIE on study performance. For instance, CRIE had a positive relationship with study performance (H4b +). However, it was also hypothesized in this study that such pedagogy would decrease “silence as a sign of power” (H4a −) which, in turn, was predicted to have a positive relationship with study performance (H3b +). The logic behind this proposition was that the direct effect of culturally responsive pedagogy on study performance was positive (H4b +), but culturally responsive pedagogy also had an indirectly negative effect on study performance through the channel of decreasing “silence as a sign of power” (H4a × H3b). In the same vein, culturally responsive pedagogy had an indirectly positive effect on study performance through the channel of decreasing “silence as a form of protection” (H4a × H2b). A similar analysis could be applied for the directly positive effects (H1) and indirectly negative effects of cultural distance on study performance, that is, H2a × H2b and H3a × H3b. The characteristics of the sample that facilitated the tests and the variable measures used in the tests are described below.

## 3. Empirical Model and Data

### 3.1. Empirical Model 

To investigate the impact of the cultural distance and classroom silence of migrant students as well as CRIE on their study performance, following the theoretical framework described in Figure 1, we used an empirical model, as follows:

(1) $${SP}_{i}=\beta_{0}+\beta_{1}CD_{i}+\beta_{2}{CSP1}_{i}+\beta_{3}{CSP2}_{i}+\beta_{4}{CRIE}_{i}+\beta_{5}X_{i}+\varepsilon_{i}$$

where SP_i_ was the study performance of a student i; the main explanatory variables included the four treatment variables of the culture distance of the students (CD_i_), classroom silence—protection (CSP1_i_), classroom silence—power (CSP2_i_), and teachers’ culturally responsive and inclusive education (CRIE_i_); X was the vector of control variables including the gender dummy (female = 1 and male = 0), three grade dummies (using the freshman grade as the baseline group), and eight subject dummies (management, law, literature, science and mathematics, technology and engineering, medical science, art, and others; using economics as the baseline group); and ε_i_ was the usual error term. H1 (β_1_ > 0), H2b (β_2_ < 0), H3b (β_3_ > 0), and H4b (β_4_ > 0) were tested by using Equation (1) for their expected signs of coefficients. At the same time, to investigate the impact of the cultural distance of migrant students and CRIE on classroom silence, we used an empirical model, as follows:

(2) $${CSP1}_{i}=\alpha_{0}+\alpha_{1}{CD}_{i}+\alpha_{2}C{RIE}_{i}+\alpha_{3}Y_{i}+\sigma_{i}$$

(3) $${CSP2}_{i}=\gamma_{0}+\gamma_{1}{CD}_{i}+\gamma_{2}C{RIE}_{i}+\gamma_{3}Y_{i}+\sigma_{i}$$

where the dependent variables were the two kinds of classroom silence (CSP1_i_ and CSP2_i_) of a student i; the main explanatory variables included the culture distance of the students (CD_i_) and the variable of culturally responsive and inclusive education (CRIE_i_); Y was the vector of control variables as above; and σ_i_ was the usual error term. H2a (α_1_ > 0), H3a (γ_1_ < 0), and H4a (α_2_ < 0, γ_2_ < 0) were tested by using Equations (2) and (3) for their expected signs of coefficients. For simplicity, the subscript i was dropped in the following sections. 

### 3.2. Data Description 

The quantitative evidence used to conduct the investigation was sourced primarily from a survey (“The Questionnaire on Classroom Silence in Shanghai 2022”) on 1051 college students in Shanghai. The data sampling was enforced through a professional web-based survey platform: WenJuanXing [88,89]. This platform was run by the largest online investigation company with a dataset covering more than 300 million potential respondents. Data were collected randomly online through widely used applications such as Sina Weibo, Webchat, and Tencent QQ. All colleges in Shanghai were covered in this sampling, so the distribution of students’ gender, subjects, and grades was consistent with the government statistics at the macro level. In total, 4092 questionnaires were sent to randomly selected college students in Shanghai and 1051 reliable responses were received. The sample period was from 23 November 2021–8 January 2022, just before the lockdown of Shanghai in March 2022. Respondents’ IP addresses could be identified to verify that most students were still studying in Shanghai when they were completing their survey questions. With the exception of the variables regarding the sociodemographic characteristics of the students, most questions were constructed by using a 1–5 scale of agreement: 1 = “Strongly disagree”, 2 = “Disagree”, 3 = “Indifference”, 4 = “Agree”, and 5 = “Strongly agree”. We discussed the measurement of the main variables of the empirical specification below, as more descriptive statistics of the variables are presented in Table 1.

#### 3.2.1. Dependent Variable 

The following sections describe the measures and scale-development procedures for the dependent variables: study performance and classroom silence. We first measured the study performance in Equation (1). There was little or no agreement on how to measure the performance of students in the literature. The selection of the measurement of study performance was based on the type of research questions being pursued. In this paper, a 1–5 scale variable of agreement (SP) was used to measure students’ subjective assessment of their study performance, that is, perceived learning gains: “Overall, my learning gains (knowledge, ability, accomplishment, etc.) are very rich.” In Table 1, the average SP was only approximately 2.23, with a standard deviation of 0.77, suggesting low average perceived learning gains of college students in Shanghai. Furthermore, the average value adding q standard deviation was only 3 (=2.23 + 0.77, “Indifference”), suggesting more than 80% of students felt unhappy with their learning gains in class. This also supports the necessity to improve the teaching quality in Chinese higher education. 

Next, we measured two types of classroom silence by using the common factor analysis (CFA) method. Our questionnaire survey gathered evidence regarding classroom silence that has been identified in the literature [2,5,6,8,33]. This relates to the areas of classroom silence as a form of protection (CSP1) and as a sign of power (CSP2). The questions that covered these two types of classroom silence may have been subject to measurement errors and would also inevitably be correlated to each other. To address these risks, we used the CFA method to obtain two common factors from the relevant questions on classroom silence in the survey. We also examined the sampling adequacy using the Kaiser–Meyer–Olkin (KMO) test. All KMO measures were more than 83% and the overall KMO was more than 86%, so we can conclude that the variables have warranted the sampling adequacy of using a CFA model. In order to keep the common factors consistent with the original 1–5 scale used for the separate variables in the survey, they were rescaled with a mean of 0 and a standard deviation of 2.

The first common factor was based on variables relating to how students feel about speaking in class. Key questions here concerned the extent to which the students used silence to protect themselves when they experienced difficulties in participating in a class discussion. The respondents were asked to indicate, on the 1–5 scale of agreement, their responses to the following four statements: (1) I feel nervous and anxious to speak in class (snervous); (2) I tend to sit in the back row or in the corner during class, not wanting to speak (scorner); (3) speaking in public makes me feel embarrassed in class (sshy); (4) I would give up asking questions because I was worried that I was stupid (sstupid). The argument of “social background and ethnic disadvantage” for migrant students posits that cultural distance, language constraints, education institutional structure, and social background differences can lead to more classroom silence as a form of protection, resulting in low academic achievement [71,72,73,74]. Higher values represent more classroom silence as a form of protection. These variables had average values around 3, with a standard deviation of 1.3 in Table 1, suggesting that more than half of the students used classroom silence as a form of protection. More than 15% of the students reported seriously negative feelings of nervousness, anxiousness, embarrassment, frustration, resistance, and alienation in class and tried to use classroom silence as a way of hiding and for self-protection. The second common factor was based on variables relating to how students used classroom silence as a sign of power. Key questions here concerned the extent to which the students used silence to influence other people in the classroom and increase the power of their subsequent speaking and participation in the class. The respondents were asked to indicate their agreement, on a scale of 1–5, with the following four statements: (1) if I question the teacher’s point of view, it will affect the teacher’s authority (stshame); (2) if I speak often, it makes people think that I am a pushy person (sostentious); (3) in class, questioning classmates’ perspectives can make classmates embarrassed (ssshame); (4) in class, I believe that “silence is golden” (ssilence). Bosacki [8] regarded classroom silence as a positive factor for adolescents’ cultural identity and self-development. The argument of “immigration optimism” addressed the positive influence of migrant values, culture, optimism, and resilience on educational outcomes [54,67,68,69,70]. The values, culture, optimism, and resilience of migrant students brings with it a better position than for native students in terms of educational success. Higher values represented more classroom silence as a sign of power. These variables had average values around 3.3, with a standard deviation of 1.2, in Table 1, suggesting that more than half of the students used classroom silence as a speaking strategy to influence teachers and peers. More than 15% of the students reported strongly agreeing that classroom silence can be used as a sign of power to strengthen their influence and cultural identity in class. 

#### 3.2.2. Main Explanatory Variables

Our sample included 131 local college students (12.4%) and 920 migrant students (87.6%) in Shanghai; of these, 394 migrant students (38%) were from northern China and 522 migrant students (49.6%) were from southern China. The cultural distance of migrant college students was determined as the south-rice-north-wheat cultural difference between the migrant college students’ hometowns and Shanghai in 1916 traditional China (CD). Based on the work of Perkins [34], the traditional south-rice-north-wheat culture was measured as the proportion of rice-planting areas in 1916 traditional China, i.e., rice% = (rice planting area)/((rice planting area + wheat planting area)) in provinces where the migrant students were born (hometown, [38,39]). Due to climate change and the technological progress in recent decades, the rice and wheat farming areas are now more interchangeable; thus, the relationship between cultural distance and study performance is likely to be subject to simultaneity or reverse causality problems, especially after industrialization. Only when the traditional agriculture sector was still dominant in the 1916 Chinese economy could the rice farming proportion be considered a suitable exogenous indicator of culture [40,41].

The Hukou system or linguistic differences were used as measure of cultural distance [90,91,92]. However, the Hukou system or linguistic differences are very difficult to measure in a continuously quantitative way. In most cases, the Hukou system and linguistic differences were measured using dummy (binary) variables, which is not a continuous quantitative measurement of cultural distance in terms of south-rice-north-wheat regions. Second, all migrant college students in Shanghai were temporarily provided Shanghai urban Hukou during their enrollment period even if they were from a rural area. Hukou has no real and tangible effect on migrant college students’ day-to-day lives as migrant workers [92]. Third, Shanghainese is a dialect of Wu Chinese, which is not mutually intelligible with Mandarin or other Chinese dialects [90,91]. However, Mandarin is the official teaching language in Chinese universities and colleges. Linguistic differences on campus are too small to have a real and tangible effect on migrant college students’ day-to-day lives as migrant workers either [92]. 

Following the same vein of Chaganti and Sambharya [93] and Krishnan et al. [94], the cultural distance was measured as the absolute value of the difference between migrant students’ hometowns (rice%student) and the host city (Shanghai, rice%Shanghai = 79%): CD = |rice%student − rice%Shanghai|. If the respondents were college students born in Shanghai, the cultural distance of local students would be zero (CD = 0). Table 1 shows that the mean value of the cultural distance was approximately 30% (with a standard deviation of 27%), suggesting a high level of cultural distance between migrant students’ hometown culture and the local culture of Shanghai. In order to test the operationalization of the south-rice-north-wheat culture in today’s China, Talhelm, Zhang, and Oishi [36] had performed psychological experiments in Starbucks coffee shops in six of China’s most modern cities: Beijing (wheat), Shenyang (wheat), Shanghai (rice), Nanjing (rice), Guangzhou (rice), and Hong Kong (rice). By observing people sitting in cafes and moving chairs together in Starbucks, the experiments proved that rice and wheat cultural heterogeneity persists in everyday life in China, suggesting the feasibility of using south-rice-north-wheat culture as a way to operationalize “culture” in modern China.

Moreover, instead of using a single indicator, we applied principal component analysis (PCA) to combine five pedagogical indicators into a composite measure of teachers’ culturally responsive and inclusive education (CRIE). The respondents were asked to indicate their agreement, on a 1–5 scale, with the following statements regarding the efforts of teachers towards culturally responsive and inclusive education: (1) teachers use interactive teaching methods to encourage students to express their views (*tinter*); (2) teachers actively communicate with students, understand students’ opinions and provide feedback (*tcommu*); (3) teachers show tolerance and provide encouragement when students fail to answer (*tencou*); (4) teachers are heuristic and can make it attractive for students to participate in an interaction (*theur*); (5) teachers are knowledgeable, charming, and willing to participate in class discussion (*stcharm*). We also examined the sampling adequacy of the five indicators using the KMO test. All KMO measures were more than 70% and the overall KMO was more than 75%, so we can conclude that these variables warranted the sampling adequacy of using a PCA model.

These variables had average values around 2.1, with a standard deviation of 1, in Table 1. This suggests that more than half of the students were unsatisfied with the efforts of teachers in terms of CRIE. More than 15% of students strongly agreed that there was no effort from teachers in terms of CRIE. This also reflects that the college classrooms in Shanghai have a lack of CRIE, which partially explains the pervasive classroom silence and students’ perceived unsatisfactory learning gains in higher education in Shanghai. In order to keep the variable factor of CRIE consistent with the original 1–5 scales used for the separate variables in the survey, CRIE was also rescaled with a mean of 0 and a standard deviation of 2. Higher values in this variable factor represent the additional efforts of teachers in terms of providing culturally responsive and inclusive education.

#### 3.2.3. Control Variables

Regarding the personal characteristics of college students, there were more female respondents in the sample (68%). With the exception of under-sampling of first-year college students who were more reluctant to be surveyed (6%), the other three grades were basically equally distributed. As an international financial and innovation center in China, approximately 39% of college students studied the subjects of economics and management, while approximately 37% of college students studied the subjects of science, technology, engineering, and mathematics (STEM) in Shanghai. These personal characteristics were controlled for all empirical analyses.

## 4. Empirical Results and Discussion

### 4.1. Study Performance

The stepwise estimation results of Equation (1) are shown in Table 2. The results presented in Column 1 show that a 1 point increase in students’ cultural distance could improve their perceived learning gains by 0.248. Hence, one standard deviation increase in migrant students’ cultural distance (0.27, see Table 1) could improve their subjective study performance by approximately 9% (=0.27 × 0.248/0.77, see Table 1) standard deviation. When we added control variables to the regression, the effect of cultural distance on perceived learning gains was still approximately 0.2 and significantly positive, supporting Hypothesis 1. The “immigration optimism” argument was supported by the quite large and positive effect of cultural distance on perceived learning gains. Migrant-specific resources, such as values, culture, optimism, and resilience, may positively influence educational outcomes. Local higher education can provide migrant students with higher perceived learning gains, which can help them participate in the economic, social, and civic life of their communities, and promote their social and emotional well-being during the later transition from college education to career development [14,92]. Hence, it confirms the optimistic view that migrant students with a larger cultural distance can benefit more from higher education in the most developed city in China. 

On the contrary, classroom silence as a form of protection significantly decreased students’ perceived learning gains in Column 3 (−0.084). That is, a 1 standard deviation increase in classroom silence as a form of protection (2, see Table 1) decreased their subjective study performance by approximately 22% (=−2 × 0.084/0.77, see Table 1) standard deviation. This was quite a large effect, and it was not very surprising to find that a lack of class participation would negatively affect the students’ perceived learning gains of knowledge, ability, accomplishment, etc., supporting Hypothesis 2b. Migrant students experiencing resistance and alienation find it more difficult to communicate with teachers and peers. They need more time to understand the teaching content with cultural contexture and interact with teachers and classmates. Thus, migrant students with classroom silence often lose the opportunity to participate in class discussion and may, therefore, display inferior study performance. 

The effect of classroom silence as a sign of power has been estimated and presented in Column 4 of Table 2. As Hypothesis 3b predicted, classroom silence can be used as a strategy to strengthen a student’s power, cultural identity, and influence in class discussions, which can have a significantly positive effect on study performance (0.019). Hence, 1 standard deviation increase in classroom silence as a sign of power (2, see Table 1) can increase their perceived learning gains by approximately 5% (=2 × 0.019/0.77, see Table 1) standard deviation. Although the effect size was not very large, the empirical evidence of the positive effect of classroom silence on perceived learning gains was significant, which was consistent with the work of Hanh [80], Harumi [79], and Maher and King [96]. This type of silence can be a sign of power in situations where students can use silence preceding speaking to influence teachers and peers. Especially for complicated discussion topics, it is not surprising that silence in power can push the difficult class discussion deeper and bring better perceived learning gains.

The final column presents the effect of teachers’ effort on study performance. Students’ perceived learning gains were much better if their teachers were more culturally responsive and inclusive (0.119). The effect size of CRIE was the largest among the main explanatory variables. A 1 standard deviation increase in CRIE (2, see Table 1) could increase their perceived learning gains by approximately 31% (=2 × 0.119/0.77, see Table 1) standard deviation. As teachers have interactive, communicative, and heuristic pedagogy, are more tolerant and encouraging to students, and show knowledgeable and charming personal characteristics, their teaching can be regarded as being more culturally responsive and inclusive. This research highlighted the significantly positive role of teachers’ effort in the study performance of the class. Moreover, most control variables had no significant effect on subjective study performance. For example, the perceived learning gains of female students were not significantly different from males. The only significant differences were found in sophomore students and literature students, who had a better assessment for their perceived learning gains than those in other grades and studying other subjects.

### 4.2. Classroom Silence

Table 3 reports the findings of our tests in relation to the additional hypotheses on classroom silence. Hypothesis 2a concerned the relationship between cultural distance and classroom silence as protection (CSP1). The findings in the first column showed that migrant students with a larger cultural distance were more likely to be silent to protect themselves in the classroom (0.425). Hence, a 1 standard deviation increase in migrant students’ cultural distance (0.27, see Table 1) could increase classroom silence as a form of protection by approximately 5.7% (=0.425 × 0.27/2, see Table 1) standard deviation. Adding more control variables in Column 2 would not change this significantly positive association. Schultz [2,5]) provided examples that migrant students, such as those who were African American and Japanese American, chose silence as a way to hide their cultural identities, beliefs, and family background. Hence, Hypothesis 2a was confirmed, i.e., cultural distance is an important determinant of classroom silence to protect students from nervousness, anxiousness, embarrassment, frustration, resistance, and alienation in class. The argument for “social background and ethnic disadvantage” for migrant students was confirmed. Migrant students facing language constraints, education institutional structure, and social background differences might exhibit more classroom silence as protection and lower academic achievement as a result. 

**Figure 2:**
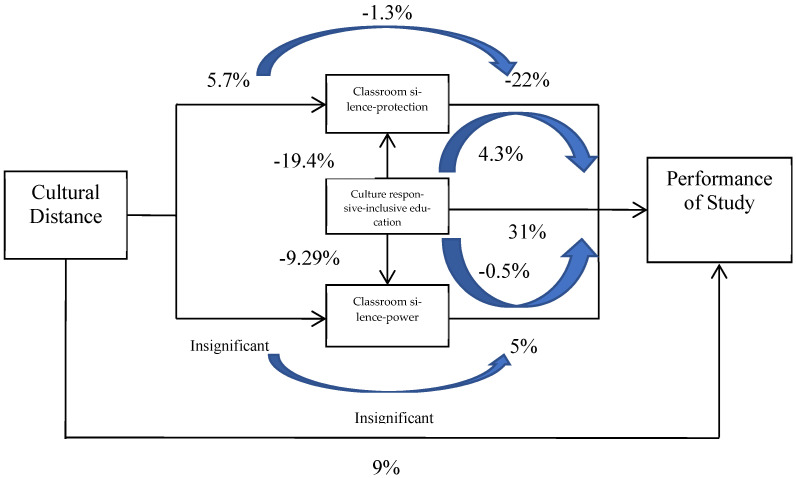
Simulation of the direct and indirect effects of cultural distance and CRIE on study performance (% standard deviation).

Meanwhile, cultural distance had an indirectly negative effect on perceived learning gains. That is, a 1 standard deviation increase in migrant students’ cultural distance could increase classroom silence as protection by approximately 5.7% standard deviation, which is associated with a 1.3% (=−5.7% × 22%, see above) standard deviation decrease in perceived learning gains. Thus, the directly positive effect of cultural distance (9%, see above) could outweigh its indirectly negative effect (−1.3%) through the channel of classroom silence as protection. The “immigration optimism” argument still outweighs the argument for “social background and ethnic disadvantage” here. The simulation of the direct and indirect effects is presented in Figure 2. 

Teachers’ culturally responsive and inclusive pedagogy can obviously alleviate this kind of negative classroom silence, as shown in column 3 (−0.194), supporting Hypothesis 4a. Combining the negative effect of classroom silence in terms of protection on study performance (Hypothesis 2b), teachers’ culturally responsive and inclusive pedagogy can improve students’ perceived learning gains by alleviating the negative effect of migrant students’ classroom silence as a form of protection. A 1 standard deviation increase in CRIE (2, see Table 1) could decrease classroom silence as a form of protection by approximately 19.4% standard deviation (=−2 × 0.194/2, see Table 1), which could then increase their subjective study performance by approximately 4.3% (=19.4% × 22%, see above) standard deviation. Therefore, 1 standard deviation increase in CRIE not only has directly positive effect on the subjective study performance of students (31% standard deviation) but could also have an indirectly positive effect (4.3% standard deviation) through the channel of decreasing classroom silence as a form of protection. 

However, this mechanism cannot be found in classroom silence as a sign of power (CSP2). First of all, cultural distance had no significant effect on this type of classroom silence. Hypothesis 3a was not supported by empirical evidence. This suggests that migrant students can also use silence as a strategy to hold up their participation in class discussions and strengthen the power and influence of their statements. It is consistent with the anecdotal evidence found by Schultz [5] highlighting that migrant students, such as those who were Mexican American, can use silence to enact a powerful stance in their classroom. Combining the directly positive effect of cultural distance (9%) with the indirectly negative effect (−1.3%) through CPS1 and the insignificantly indirect effect through CPS2, the “immigration optimism” argument is still dominant.

Second, teachers appeared to be unable to clearly identify the meanings of classroom silence (protection or power) as they tried to break both kinds of classroom silence by using culturally responsive and inclusive pedagogy. Hence, Hypothesis 4a still holds for classroom silence as a sign of power (−0.092). A 1 standard deviation increase in CRIE (2, see Table 1) could decrease classroom silence as a sign of power by approximately 9.2% standard deviation (=−2 × 0.092/2, see Table 1), which could then decrease their subjective study performance by approximately 0.5% standard deviation (=9.2% × 5%, see above). The indirectly negative effect of CRIE was actually negligible through the channel of decreasing classroom silence as a sign of power.

Last but not least, since classroom silence as a sign of power had a significantly positive effect on study performance (Hypothesis 3b), teachers’ culturally responsive and inclusive pedagogy indirectly contained students’ study performance by forcing students to break classroom silence as a sign of power. Even though the cultural distance of migrant students could not prevent them from applying strategic silence as a power strategy, the inaccurate CRIE might have decreased their perceived learning gains. Therefore, combining the directly positive effect of CRIE (31%) with the indirectly positive effect (4.3%) through CPS1 and indirectly negative effect through CPS2 (−0.5%), the positive effect of CRIE on migrant students’ learning performance was still dominant. Teachers’ CRIE played the most important role in migrant students’ perceived learning gains.

## 5. Conclusions and Discussion

Classroom silence is a pervasive phenomenon in education, especially for those students in a transitional period, such as migrant college students in a large city far from their hometowns. This paper investigated the relationship between cultural distance and classroom silence in college students in Shanghai using a dataset from 1051 college students who were surveyed using “The Questionnaire on Classroom Silence in Shanghai 2022”. We found a significantly positive association between migrant students’ cultural distance and their perceived learning gains in class, which is consistent with the argument of “immigration optimism” [54,67,68,69,70]. Moreover, there was a significantly positive association between cultural distance and classroom silence as a form of protection, but not for classroom silence as a sign of power. Classroom silence as protection decreased students’ assessment of their perceived learning gains in class, such as knowledge, ability, accomplishment, etc. However, classroom silence can be used by both local and migrant students as a hold-up strategy to strengthen their power and influence in a class discussion, which can bring more attention and engagement from teachers and peers. Thus, classroom silence as a sign of power can improve the perceived learning gains of students.

This study also highlighted the role of teachers in classroom silence. Culturally responsive and inclusive education (CRIE) can result in increased classroom interaction and communication, which undoubtedly improves students’ perceived learning gains. Teachers delivering CRIE were the most important determinant of migrant students’ perceived learning gains in our study. However, we also found that this positive outcome was influenced by the identification of the definitions of classroom silence. Teachers find it difficult to accurately identify the different meanings of classroom silence as a form of protection and as a sign of power. When teachers express frustration with silent students, they often fail to recognize how silence might be connected more broadly to a larger set of interactions in the classroom. Thus, a cautious identification of classroom silence will improve the effectiveness of culturally responsive and inclusive pedagogy. 

Based on the above comprehensive results, this study has pedagogical implications and provides suggestions for educators. When students possess a diverse cultural background, culturally responsive strategies can have important benefits for their integration into classroom instruction. Teachers should encourage students to draw on their cultural background in order to contribute to class discussions, providing an anchor for their teachers and peers to teach and learn. Hence, real understanding of students’ social and cultural backgrounds requires encouraging students to leverage their cultural capital, especially for those who do not have a voice and use silence as a form of protection. It is very important for those in a cultural minority in a mixed classroom to feel that they are an expert and have power. When silence for protection has been transformed into silence and speaking for power, teachers and peers can draw from their experiences and learn more from their culture. 

We also have suggestions for readers and future researchers related to our findings. First, this study attempted to approach the topics of migration and cultural distance that can take place within a nation, in this case, China. Although this approach has the potential to offer solid contributions to the research literature going forward, the uncritical comparison and contrast of migration that happens within a nation to international migration requires the attention of readers and future researchers. Dealing with diversity in education has been approached in multiple ways around the world. Even with the rapid globalization process, a given ideology relates to a specific context, especially in economic–political institutions. Drawing similarities between the current subnational migrant case and some of the international migrant literature that is used to support studies requires additional caveats. Future researchers should always take into account the societal and historical contexts in which earlier studies are carried out, and the validity of the current application. 

Second, culturally responsive and inclusive education (CRIE) in a Western context can be problematic in the Chinese context since it discusses issues of ethnic diversity, which are not the core issues in Chinese CRIE. With the idea of “education for all”, China has the world’s most extensive regime of minority affirmative action policies, or “preferential policies”, in terms of admission to educational institutions for ethnic minorities [17]. The decade-long scholarship on Chinese CRIE, locally and internationally, is also a testimony to China’s serious interest in education equality. Readers and future researchers should be reminded that, in our survey, students perceived incapability in their teachers to identify differences in silence. More research is needed from the teachers’ and governmental administrative perspectives to compare the results and reach conclusions in a non-Western context. 

Finally, in order to ensure that teaching is more culturally responsive, teachers should establish a student-centered education concept to break classroom silence and improve the quality of undergraduate teaching in Chinese higher education. Teachers also need to develop cross-cultural teaching methods by using more interactive, communicative, and heuristic pedagogy, and to show knowledgeable and charming personal characteristics which can help them to accurately identify the different meanings of classroom silence. The effectiveness of school and community policy should be based on eliminating discrimination and facilitating equality. Schools should improve their management system in a manner which is beneficial to students’ learning, so that classroom silence can be broken in a definite direction. Thus, there is no doubt that more evidence-based research is needed in the future to identify and promote culturally responsive and inclusive strategies in schools and communities so that policy and practice can effectively support students during their transitions throughout their life.

## Figures and Tables

**Table 1:** Descriptive statistics of variables, 1051 college students in Shanghai.

Variable	Description	Mean	Std. Dev.	Min	Max
Dependent variables					
SP	My learning gains (knowledge, ability, accomplishment, etc.) are rich	2.23	0.77	1	5
CSP1	Factor, higher value = high classroom silence (protection)	0.00	2.00	−4.84	4.76
*snervous*	I feel nervous and anxious to speak in class	2.72	1.31	1	5
*scorner*	I tend to sit in the back row or in the corner during class, not wanting to speak	3.10	1.34	1	5
*sshy*	Speaking in public makes me feel embarrassed in class	2.86	1.28	1	5
*sstupid*	I would give up asking questions because I was worried that I was stupid	2.89	1.32	1	5
CSP2	Factor, higher value = high classroom silence (power)	0.00	2.00	−6.32	6.07
*stshame*	If I question the teacher’s point of view, it will affect the teacher’s authority	3.58	1.12	1	5
*sostentious*	If I speak a lot, it makes people think that I am a pushy person	3.15	1.31	1	5
*ssshame*	In class, questioning classmates’ perspectives can make classmates embarrassed	3.14	1.18	1	5
*ssilence*	In class, I believe that “silence is golden”	3.46	1.17	1	5
Main explanatory variables					
CD	Cultural distance = |rice%student − rice%Shanghai (79%)|	0.30	0.27	0.00	0.79
CRIE	Factor, higher value = more culturally responsive, inclusive teaching	0.00	2.00	−3.47	6.28
*tinter*	Teachers use interactive teaching methods to encourage students to express their views	2.25	1.05	1	5
*tcommu*	Teachers actively communicate with students, understanding students’ opinions and providing feedback	2.47	1.10	1	5
*tencou*	Teachers show tolerance and provide encouragement if students fail to answer	1.96	0.95	1	5
*theur*	Teachers are heuristic and can make it attractive for students to participate in an interaction	2.48	1.07	1	5
*stcharm*	Teachers are knowledgeable, charming, and willing to participate in class discussion	1.82	1.01	1	5
Control dummy variables					
Gender	1 = female; 0 = male	0.68	0.47	0	1
g1	Freshman	0.06	0.24	0	1
g2	Sophomore	0.27	0.44	0	1
g3	Junior	0.32	0.47	0	1
g4	Senior	0.36	0.48	0	1
s1	Economics	0.17	0.38	0	1
s2	Management	0.22	0.41	0	1
s3	Law	0.03	0.18	0	1
s4	Literature	0.10	0.30	0	1
s5	Science and mathematics	0.11	0.31	0	1
s6	Technology and engineering	0.19	0.39	0	1
s7	Medical science	0.07	0.26	0	1
s8	Art	0.05	0.21	0	1
s9	Others (philosophy, education, history, agriculture, military science)	0.07	0.25	0	1

Data source: “The Questionnaire on Classroom Silence in Shanghai 2022”.

**Table 2:** Estimated effects of cultural distance, classroom silence, and CRIE on study performance by using Equation (1).

Dependent Variable: Study Performance (SP)	(1)	(2)	(3)	(4)	(5)
Cultural distance	0.248 ***	0.210 **	0.246 ***	0.245 ***	0.162 **
	***(0.087)***	***(0.088)***	***(0.086)***	***(0.086)***	***(0.082)***
CSP1			−0.084 ***	−0.085 ***	−0.062 ***
			***(0.012)***	***(0.012)***	***(0.011)***
CSP2				0.019 *	0.030 ***
				***(0.012)***	***(0.011)***
CRIE					0.119 ***
					***(0.011)***
Female		−0.026	−0.067	−0.075	−0.057
		***(0.053)***	***(0.052)***	***(0.052)***	***(0.049)***
Sophomore		0.232 **	0.225 **	0.236 **	0.184 *
		***(0.106)***	***(0.104)***	***(0.104)***	***(0.099)***
Junior		0.008	0.024	0.033	0.000
		***(0.105)***	***(0.102)***	***(0.102)***	***(0.097)***
Senior		0.051	0.019	0.026	−0.054
		***(0.104)***	***(0.102)***	***(0.102)***	***(0.097)***
Management		0.107	0.118	0.117	0.095
		***(0.077)***	***(0.075)***	***(0.075)***	***(0.072)***
Law		0.116	0.102	0.094	0.073
		***(0.141)***	***(0.137)***	***(0.137)***	***(0.131)***
Literature		0.269 ***	0.251 ***	0.251 ***	0.282 ***
		***(0.095)***	***(0.093)***	***(0.093)***	***(0.088)***
Science and mathematics		0.108	0.079	0.077	0.063
		***(0.093)***	***(0.091)***	***(0.091)***	***(0.086)***
Technology and engineering		0.118	0.084	0.080	0.048
		***(0.080)***	***(0.079)***	***(0.079)***	***(0.075)***
Medical science		−0.017	−0.022	−0.026	−0.025
		***(0.106)***	***(0.103)***	***(0.103)***	***(0.098)***
Art		0.166	0.151	0.151	0.150
		***(0.123)***	***(0.120)***	***(0.120)***	***(0.114)***
Others		0.160	0.118	0.123	0.095
		***(0.107)***	***(0.104)***	***(0.104)***	***(0.099)***
R-squared	0.008	0.032	0.078	0.080	0.168
N	1051	1051	1051	1051	1051

Note: *, **, and *** represent the significance levels of 10%, 5%, and 1%, respectively. The italic and bold numbers in brackets below the coefficients represent the standard deviations of the estimated coefficients. All analyses were performed using Stata 17 [95].

**Table 3:** Estimated effects of cultural distance and inclusive education on classroom silence by using Equations (2) and (3).

Dependent Variable: Classroom Silence (CS)	CSP1			CSP2		
	(1)	(2)	(3)	(4)	(5)	(6)
Cultural distance	0.425 *	0.423 *	0.540 **	0.071	0.070	0.126
	***(0.227)***	***(0.227)***	***(0.224)***	***(0.227)***	***(0.230)***	***(0.230)***
Culturally responsive			−0.194 ***			−0.092 ***
			***(0.030)***			***(0.031)***
Female		−0.481 ***	−0.500 ***		0.430 ***	0.422 ***
		***(0.136)***	***(0.134)***		***(0.138)***	***(0.137)***
Sophomore		−0.089	0.009		−0.572 **	−0.526 *
		***(0.274)***	***(0.270)***		***(0.278)***	***(0.277)***
Junior		0.186	0.241		−0.466 *	−0.440
		***(0.270)***	***(0.265)***		***(0.273)***	***(0.272)***
Senior		−0.371	−0.220		−0.336	−0.264
		***(0.269)***	***(0.265)***		***(0.272)***	***(0.272)***
Management		0.134	0.162		0.081	0.095
		***(0.200)***	***(0.196)***		***(0.202)***	***(0.201)***
Law		−0.175	−0.140		0.381	0.398
		***(0.363)***	***(0.357)***		***(0.368)***	***(0.366)***
Literature		−0.212	−0.254		0.007	-0.014
		***(0.246)***	***(0.241)***		***(0.249)***	***(0.248)***
Science and mathematics		-0.344	-0.309		0.082	0.099
		***(0.240)***	***(0.236)***		***(0.243)***	***(0.242)***
Technology and engineering		-0.406 *	-0.341 *		0.186	0.217
		***(0.207)***	***(0.204)***		***(0.210)***	***(0.209)***
Medical science		−0.056	−0.057		0.190	0.189
		***(0.273)***	***(0.268)***		***(0.276)***	***(0.275)***
Art		−0.180	−0.172		−0.013	−0.009
		***(0.318)***	***(0.312)***		***(0.322)***	***(0.321)***
Others		−0.499 *	−0.429		−0.258	−0.225
		***(0.276)***	***(0.271)***		***(0.279)***	***(0.278)***
R-squared	0.003	0.039	0.076	0.000	0.017	0.026
N	1051	1051	1051	1051	1051	1051

Note: *, **, and *** represent the significance levels of 10%, 5%, and 1%, respectively. The italic and bold numbers in brackets below the coefficients represent the standard deviations of the estimated coefficients.

## Data Availability

The data presented in this study are available on request from the corresponding author. The data are not publicly available due to privacy.
